# Explaining Unsaturated Fatty Acids (UFAs), Especially Polyunsaturated Fatty Acid (PUFA) Content in Subcutaneous Fat of Yaks of Different Sex by Differential Proteome Analysis

**DOI:** 10.3390/genes13050790

**Published:** 2022-04-28

**Authors:** Lin Xiong, Jie Pei, Xiaoyun Wu, Pengjia Bao, Xian Guo, Ping Yan

**Affiliations:** 1 Animal Science Department, Lanzhou Institute of Husbandry and Pharmaceutical Sciences, Chinese Academy of Agricultural Sciences, Lanzhou 730050, China; xionglin@caas.cn (L.X.); peijie@caas.cn (J.P.); wuxiaoyun@caas.cn (X.W.); baopengjia@caas.cn (P.B.); guoxian@caas.cn (X.G.); 2 Key Laboratory for Yak Genetics, Breeding, Reproduction Engineering of Gansu Province, Lanzhou 730050, China

**Keywords:** yak, subcutaneous fat, proteome, UFAs and PUFAs

## Abstract

Residents on the Tibetan Plateau intake a lot of yak subcutaneous fat by diet. Modern healthy diet ideas demand higher unsaturated fatty acids (UFAs), especially polyunsaturated fatty acid (PUFA) content in meat. Here, the gas chromatography (GC) and tandem mass tag (TMT) proteomic approaches were applied to explore the relationship between the proteomic differences and UFA and PUFA content in the subcutaneous fat of yaks with different sex. Compared with male yaks (MYs), the absolute contents of UFAs, monounsaturated fatty acids (MUFAs) and PUFAs in the subcutaneous fat of female yaks (FYs) were all higher (*p* < 0.01); the relative content of MUFAs and PUFAs in MY subcutaneous fat was higher, and the value of PUFAs/SFAs was above 0.4, so the MY subcutaneous fat is more healthy for consumers. Further studies showed the transcriptional regulation by peroxisome proliferator-activated receptor delta (PPARD) played a key role in the regulation of UFAs, especially PUFA content in yaks of different sex. In FY subcutaneous fat, the higher abundance of the downstream effector proteins in PPAR signal, including acyl-CoA desaturase (SCD), elongation of very-long-chain fatty acids protein 6 (ELOVL6), lipoprotein lipase (LPL), fatty acid-binding protein (FABP1), very-long-chain (3R)-3-hydroxyacyl-CoA dehydratase 3 (HACD3), long-chain fatty acid CoA ligase 5 (ACSL5) and acyl-CoA-binding protein 2 (ACBP2), promoted the UFAs’ transport and synthesis. The final result was the higher absolute content of c9-C14:1, c9-C18:1, c9,c12-C18:2n-6, c9, c12, c15-C18:3n-3, c5, c8, c11, c14, c17-C20:5n-3, c4, c7, c10, c13, -c16, c19-C22:6n-3, UFAs, MUFAs and PUFAs in FY subcutaneous fat. Further, LPL, FABP1, HACD3, ACSL1 and ACBP2 were the potential biomarkers for PUFA contents in yak subcutaneous fat. This study provides new insights into the molecular mechanisms associated with UFA contents in yak subcutaneous fat.

## 1. Introduction

Consumers are paying more and more attention to fat intake, nowadays [1]. Dietary fatty acids directly influence human health, and have been associated with obesity, non-alcoholic fatty liver and other diseases [2]. Monounsaturated fatty acids (MUFAs) and polyunsaturated fatty acids (PUFAs), especially n-3 PUFAs, are beneficial to cardiovascular health [3]. Carcass fat is deposited in different anatomical locations as subcutaneous, visceral, inter-muscular fat and intra-muscular fat depots. In previous reports, studies have mainly focused on the fatty acids in cattle intra-muscular [4] and subcutaneous fat [5]. 

Yak (*Bos grunniens*) is a unique livestock on the Tibetan Plateau and its adjacent areas, and there are now more than 15 million yaks [6]. Yak meat is the major source of animal protein in the local human diet [7], and the annual yield of yak meat is approximately 300 thousand tons [8]. The fat content in yak muscle is 0.38–5.81% [9]. Most residents on the Tibetan plateau have a short life, and the average lifetime of residents is less than 60 years at present. The harsh environment here, including low temperature and oxygen-poor, on the Tibetan plateau, has led to this situation. On the other hand, diet is another major factor leading to a short life on the Tibetan plateau. As everyone knows, a high-fat diet can lead to cardiovascular disease. A high yak meat intake and a rare vegetable intake on the Tibetan plateau can cause cardiovascular disease. The unsaturated fatty acid (UFA) and PUFA content in yak meat is closely related to cardiovascular and cerebrovascular diseases, so it is important and necessary to study the UFA and PUFA content in yak meat. Modern yak meat processing facilities are scarce on the Tibetan Plateau at present, and the food processing technologies for yak meat are still in the initial stage. Usually, yak muscle with much subcutaneous fat is cooked and consumed. By contrast, the quantity of intra-muscular fat from yak meat consumed is very limited. Therefore, the study of UFA and PUFA content in yak subcutaneous fat is more meaningful than in yak intra-muscular fat. Under the same genetic background, yaks with different UFA and PUFA content in subcutaneous fat are a good model for studying the molecular mechanism of UFA and PUFA deposition. Sex can influence the fatty acids content in cattle [10], and maybe there are differences in the UFA and PUFA content in subcutaneous fat between female (FYs) and male yaks (MYs).

Some genes regulating the fatty acids content in cattle have now been found by genomic studies for phenotypic profiling [11]. However, the same gene can produce different proteins by the alternative splicing of mRNA [12], so it is not enough to understand the regulatory mechanism of fatty acid content in cattle only by genomic studies. Proteomic studies can reveal the molecular mechanism of fatty acid metabolism from the protein level in a biological sample, and they have been applied to assessing meat sensory quality [13] and nutritional value [14]. The study of adipose tissue proteome is of great value for understanding the molecular mechanisms associated with UFA content. The UFA content in yak adipose tissue is largely determined by the key enzymes involved in UFA metabolism. In this study, the hypothesis was that the subcutaneous fat from FYs and MYs would exhibit a differential abundance of proteins regulating the UFA and PUFA content. To our knowledge, no study on exploring crucial proteins involved in UFA content in yak subcutaneous fat has been reported at present. The Tandem Mass Tags (TMT) proteomic approach possesses the potential to provide novel information with greater proteome coverage [15], and has been applied to studying fat metabolism in cattle [16,17]. 

In this study, the fatty acid content in FYs and MYs subcutaneous fat was detected by gas chromatography (GC), and then the TMT proteomic method was used to identify differentially expressed proteins (DEPs) in FYs and MYs subcutaneous fat. Further, the functional enrichment of gene ontology (GO) terms analysis was performed to determine the pathways and processes that were enriched in the differential expression profile, and the crucial DEPs and metabolic pathways involved in regulating UFA and PUFA content were screened. Finally, liquid chromatography–parallel reaction monitoring–mass spectrometry (LC-PRM-MS) was applied to verify the accuracy of the TMT proteomic method. The regulatory mechanism and potential biomarkers for UFA and PUFA content in yak subcutaneous fat were explored.

## 2. Materials and Methods

### 2.1. Animals, Diet and Samples Collection

Animal experiments and samples collection were carried out in accordance with the Guidelines for Care and Use of Laboratory Animals of China and all protocols were approved by the Institutional Review Board of the Lanzhou Institute of Husbandry and Pharmaceutical Sciences, Chinese Academy of Agricultural Sciences. The animal experiment was carried out in the pasture in Haibei, China. Six MYs (Qinghai huanhu yak, 315 ± 10 kg, 4 years old, intact) and 6 FYs (Qinghai huanhu yak, 255 ± 10 kg, 4 years old) were selected, and the two groups were isolated. All yaks were raised in the same environment with natural grazing, and had free access to grass and water with no supplementary feeding. By late September, all yaks were humanely harvested at a commercial abattoir in Haibei by electrical stunning, and the subcutaneous fat above longissimus dorsi (12–13th rib level) was collected immediately. Parts of the fat samples were placed in the enzyme-free cryopreservation tubes and kept in liquid nitrogen, and others were put into the sealed pockets and frozen at −20 °C in fridge for chemical analysis. In addition, the grass samples were collected in early September. Ten sampling points were uniformly distributed over the whole pasture. One thousand g fresh grass sample was collected at each sampling point. The common nutrition and fatty acid content in grass were detected according to China’s national standard for forage feed. A total of 18 fatty acids, including 11 saturated fatty acids (SFAs), 4 MUFAs and 3 PUFAs, were detected in grass, and C16:0, C18:0, c9-C18:1, c9, c12-C18:2n6 and c9, c12, c15-C18:3n3 were the main fatty acids. The nutrition content of grass (dry matter) is shown in Table 1.

### 2.2. Determination of Fatty Acid Content in Subcutaneous Fat

The fatty acid content in subcutaneous fat was analyzed by GC, and the operating method can be described as follows [18]. A three-gram fat sample was transferred into a mortar for grinding, and the liquid nitrogen was continuously added to the mortar to keep low temperature. Then, a 10 mL mixed solution (chloroform: methanol, *v*:*v*, 2:1) was used to extract the fat for 5 times, and the extracting solution was merged in a 100 mL conical flask. The whole solution was filtered into an evaporation flask, and dried under nitrogen. Then, 10 mg extracted fat sample and 0.1 mol/L sodium hydroxide methanol solution (2 mL) were put into a centrifuge tube, and the centrifuge tube was placed into a water bath at 60 °C for 1 h. Subsequently, 2 mL 15% boron fluoride–methanol solution was added to the mixture after it reached room temperature, and the centrifuge tube was placed into incubator shakers at 60 °C for 1 h. Then, 2 mL saturated sodium chloride solution, 2 mL n-hexane and 5 mL distilled water were added into the centrifuge tube, and the centrifuge tube was shaken in an oscillator for 10 min. Lastly, the mixture was let stand for 10 min. The supernatant was collected, and filtered into a vial using a polytetrafluoroethylene (PTFE) filter (Millipore Corp, Burlington, MA, USA). The extract was determined by a 7890A GC system (Agilent Corp., Santa Clara, CA, USA) coupled with a flame ionization detector (FID), and an Agilent W&J CP-Sil88 FAME capillary (100 m × 0.25 mm, 0.20 µm) was used to separate the extract. Analytical standards with high purity were used in the experiment, and were provided by Sigma-Aldrich Corp (Laramie, WY, USA). The GC conditions were set as follows: The injector and detector temperatures were 260 and 280 °C, respectively. The injection volume was 1 μL. The temperature ramp program was as follows: The initial column oven temperature was maintained at 100 °C for 5 min and then increased by 8 °C/min to 180 °C and held for 9 min; next, the temperature was raised to 230 °C at 1 °C/min, where it was maintained for 15 min. The nitrogen constant linear flow rate was 0.5 mL/min. The split/splitless injector was used with a split/splitless ratio of 1:100. Linearity of the FID chromatographic response was tested with six calibration points across the range 0.002–50 g/100 g. The linear regression coefficients for the calibration curves showed good results (R^2^ 0.9913–0.9990). The limit of detections (LODs) for fatty acids was in the range 0.0007–0.0028 g/100 g, and the limit of quantifications (LOQ)s was in the range 0.0020–0.0062 g/100 g. Recoveries for fatty acids were 78.82–90.06% in the inter-day experiment, and 80.60–88.65% in the intra-day experiment. The inter- and intra-day relative standard deviation (RSDs) for fatty acids ranged from 3.6% to 10.0% and 4.5% to 10.2%, respectively. The chromatogram of fatty acids in subcutaneous fat of FYs and MYs are shown in Figures S1 and S2, respectively. The fatty acids were named by the number of carbons, unsaturated bonds and the position of unsaturated bonds. The absolute content of fatty acids was obtained by comparing with the standard, and the relative content was calculated by dividing the absolute content of a certain fatty acid into the absolute content of total fatty acids. 

### 2.3. Proteomics Analyses

#### 2.3.1. Protein Extraction and Sodium Dodecyl Sulfate-Polyacrylamide Gel (SDS-PAGE)

Three subcutaneous fat samples per group were randomly selected for the following proteomic analysis. Frozen fat samples were transferred into low protein binding tubes (1.5 mL) and lysed with 300 µL lysis buffer supplemented with 300 µL 1 mmol/L phenylmethanesulfonyl fluoride (PMSF). Subsequently, the mixture was degraded by ultrasound at 80 W for 3 min, followed by centrifugation at 15,000 r/min for 15 min. Then, a 10 µg protein in fat sample was acquired and separated using 12% sodium dodecyl sulfate-polyacrylamide gel (SDS-PAGE), and the separation gel was stained by coomassie brilliant blue according to Candiano’s protocol [19]. 

#### 2.3.2. Digestion with Trypsin and Tandem Mass Tag (TMT) Labeling

The sample was dissolved in SDS Lysis Buffer in an ultrafiltration tube. The dithiothreitol (DTT) solution was added to the tube, and the final concentration of DTT was 5 mmol/L. The mixture was incubated at 55 °C for 30 min, then cooled to room temperature using ice. Subsequently, the iodoacetamide solution was added to the tube, and the final concentration of iodoacetamide was 10 mmol/L. The mixture was fully shaken and let stand for 15 min away from light. In order to precipitate protein, acetone was added to the tube, and the mixture was let stand for another 4 h at −20 °C, followed by centrifugation at 8000 r/min for 10 min. The sediment was collected, and redissolved in 100 μL tetraethyl-ammonium bromide (TEAB) solution. One mg/mL trypsinization Trypsin-TPCK was added to the mixture, and the mixture was stewed overnight. The sample, after enzymolysis, was freeze-dried and stored at −80 °C. The freeze-dried sample was dissolved in 66 μL 200 mmol/LTEAB buffer, and a 30 μL solution was transferred into an Eppendorf (EP) tube. TMT reagent was dissolved in 88 μL acetonitrile, and the solution was vortexed for 5 min, followed by centrifugation. A 41 μL solution of TMT reagent was added into the sample solution in an EP tube, and the mixture was vortexed and let stand for 1 h at room temperature. Then 8 μL 5% hydroxylamine was added to the EP tube, and the solution was freeze-dried after 15 min. Freeze-dried samples were stored at −80 °C.

#### 2.3.3. Reverse-Phase Liquid Chromatography (RPLC) Separation and Mass Spectrometry (MS) Analysis

Reverse-phase liquid chromatography (RPLC) separation was performed on an Agilent 1100 HPLC System with an Agilent Zorbax Extend RP column (5 μm, 150 mm × 2.1 mm). Mobile phases A (0.1% formic acid in water) and B (80% acetonitrile, 0.1% formic acid in water) were used for the RPLC gradient. The solvent gradient was set as follows: 0–40 min, 5–30% B; 40–54 min, 30–50% B; 54–55 min, 50–100% B; 55–60 min, 100% B. Tryptic peptides were separated at a fluent flow rate of 300 μL/min, and monitored at 210 and 280 nm. Samples were collected for 8–60 min, and eluent was collected in centrifugal tubes 1–15 every minute, in turn. Samples were recycled in this order until the end of the gradient. The separated peptides were lyophilized for mass spectrometry. Mass spectrometry (MS) analysis was performed using a Q-Exactive mass spectrometer (Thermo, Waltham, MA, USA) equipped with a Nanospray Flex source (Thermo, Waltham, MA, USA). Samples were loaded by a capillary C18 trap column (3 cm × 100 µm), and separated by a C_18_ column (15 cm × 75 µm) on an E ksigent nanoLC-1D plus system. Mobile phase was set as A (acetonitrile:formic acid:H_2_O, 2%:0.1:97.9%, *v*:*v*:*v*) and B (acetonitrile:formic acid:H_2_O, 95:0.1:4.9, *v*:*v*:*v*). The flow rate was set at 300 nL/min, and linear gradient was set as follows: 0.0–0.5 min, 95.0–92.0% A; 0.5–48.0 min, 92.0–74.0% A; 48.0–61.0 min, 74.0–62.0% A; 61.0–61.1 min, 62.0–15.0% A; 61.1–67.0 min, 15% A; 67.0–67.1 min, 15.0–95.0% A; 67.1–70.0 min, 95.0% A. Mass spectrum (MS) data were acquired with a 2.4 kV ion spray voltage, 35 psi curtain gas, 5 psi nebulizer gas, and an interface heater temperature of 150 °C. The MS was scanned between 300 and 1600 with an accumulation time of 250 ms and a mass resolution of 70,000. The ten most intense peaks in MS were fragmented with higher-energy collisional dissociation (HCD) with NCE of 32. MS/MS spectra were obtained with a resolution of 17,500 with an AGC target of 2 × 10^5^ and a max injection time of 80 ms. The Q-E dynamic exclusion was set for 30.0 s and run under positive mode.

### 2.4. Liquid Chromatography–Parallel Reaction Monitoring–Mass Spectrometry (LC-PRM-MS) Analysis

Three subcutaneous fat samples per group were selected for LC-PRM-MS analysis, respectively. The protein samples for the TMT proteomic approach were still used here. Samples were separated by the Easy nLC 1200 system (Thermo Scientific, Waltham, MA, USA) with a Home-made tip-C18 column (75 μm × 200 mm, 3 μm). MS data were obtained on Q-Exactive HF (Thermo Scientific).

### 2.5. Statistical Analysis

The absolute and relative content of fatty acids was analyzed using independent-sample T test in Statistical Package for the Social Sciences (SPSS) software version 16.0 (SPSS Inc., Chicago, IL, USA), and Pearson correlation analysis was carried out using SPSS software version 16.0. The significance threshold for the above analyzes was set at *p* < 0.05. The raw spectrum files were searched separately with ProteomeDiscoverer software version 2.4 (Thermo Fisher Scientific Inc., Waltham, MA, USA). The parameters for searching were set as follows: dynamic modification: oxidation (M), acetyl (N-term); digestion: trypsin; instrument: Q Exactive; MS1 tolerance: 10 ppm; MS2 tolerance: 0.02 Da; missed cleavages: 2; database: GCF_000298355.1_BosGRuv 2.0_protein.fasta. Proteins with *p* < 0.05 and fold change (FC) of ±2 or greater were considered to be differentially expressed proteins (DEPs). GO analysis was performed to identify DEPs significantly at *p* < 0.05. The raw data in PRM were analyzed using Skyline 3.5.0 software (MacCoss Lab, University of Washington, Seattle, WA, USA).

## 3. Results

### 3.1. Fatty Acid Content in Yak Subcutaneous Fat

A total of 43 fatty acids (24 SFAs, 8 MUFAs and 11 PUFAs) in yak subcutaneous fat were detected in this study. The rumen biohydrogenation intermediates (BHIs) are derived from dietary PUFA in ruminants, and can be transferred into subcutaneous adipose tissue. In essence, BHIs are still PUFAs. Furthermore, these fatty acids included four BHIs and seven branched-chain fatty acids (BCFA). The result of fatty acid content is shown in Table 2. The absolute content of c9-C14:1, C16:0, c9-C16:1, C18:0, c9-C18:1, c9, c12-C18:2n-6, c9, c12, c15-C18:3n-3, c5, c8, c11, c14, c17-C20:5n-3 and c4, c7, c10, c13, c16, c19-C22:6n-3 in FY subcutaneous fat was higher (*p* < 0.05), whereas the absolute content of C15:0ai and C16:0iso in FY subcutaneous fat was lower (*p* < 0.05); moreover, the absolute content of SFAs, MUFAs, PUFAs, UFAs, n-3PUFAs and n-6PUFAs in FY subcutaneous fat was higher (*p* < 0.05). On the other hand, the relative content of c9-C14:1, C18:0 and c4, c7, c10, c13, c16, c19-C22:6n-3 in FY subcutaneous fat was higher (*p* < 0.05), whereas the relative content of C15:0ai, C16:0iso, C17:0, C17:0iso, C17:0ai, c9-C18:1, t11-C18:1 and c11, c14, c17-C20:3n-3 in FY subcutaneous fat was lower (*p* < 0.05); moreover, the relative content of SFAs in FY subcutaneous fat was higher (*p* < 0.05), whereas the relative content of MUFAs, UFAs, PUFAs, BSFAs, and PUFAs/SFAs in FY subcutaneous fat was lower (*p* < 0.05).

### 3.2. Protein Quantification and Identification

**Figure 1:**
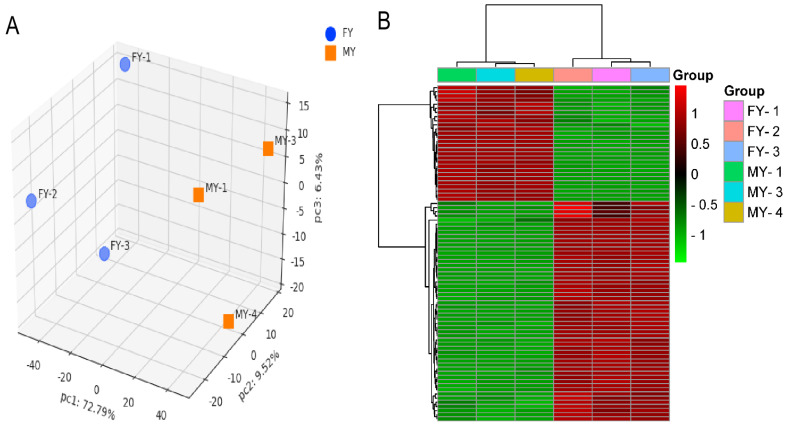
(**A**) Principal component analysis (PCA) of the subcutaneous fat in female (FYs) and male yaks (MYs); (**B**) Hierarchical clustering analysis of the proteome profiles in FYs and MYs subcutaneous fat. Red and green indicate higher and lower expression values, respectively.

Principal component analysis (PCA) score plot for subcutaneous fat samples in the FYs and MYs groups is shown in Figure 1A. Two clusters corresponding to FY and MY subcutaneous fat were clearly detectable with a sharp separation, which verified the effect of sex to the proteome composition in yak subcutaneous fat. The dendrogram of clustering analysis showed that these DEPs in subcutaneous fat could be separated from FYs to MYs completely (Figure 1B), implying that the abundance differences in the DEPs of the two groups were significant. A total of 3411 proteins were identified with at least 2 unique peptides, and in which 82 DEPs (Table S1) were obtained. Compared to the MYs group, 53 DEPs possessed a higher abundance but 29 DEPs had a lower abundance in the FYs group. The number of crucial DEPs involved in UFAs metabolism was 17, and their information is shown in Table 3. Moreover, the peroxisome proliferator-activated receptor (PPAR) is a crucial transcription factor regulating lipogenic genes in cattle [20], and there was also a difference in the abundance of peroxisome proliferator-activated receptor delta (PPARD) in subcutaneous fat between the FYs and MYs groups (Table 3).

### 3.3. Functional Enrichment Analysis of Differentially Expressed Proteins (DEPs)

**Figure 2:**
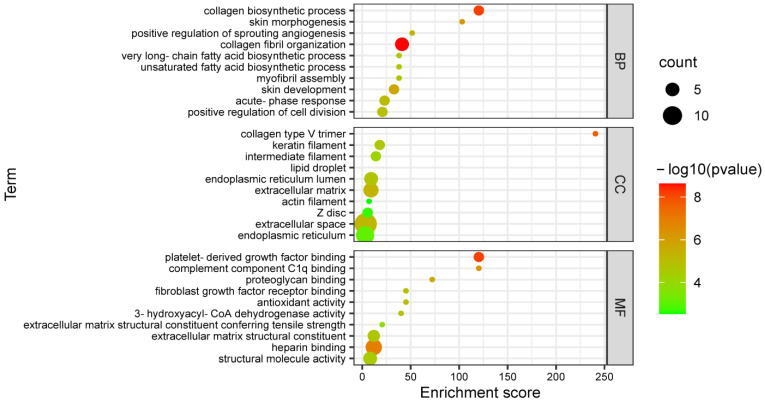
The top 30 items of Gene Ontology (GO) enrichment of differentially expressed proteins (DEPs) in FYs and MYs subcutaneous fat. BP: biological process; CC: cellular component; MF: molecular function.

To further investigate the biological processes and the molecular function associated with DEPs, GO analysis by running queries for each DEPs against the GO database was adopted, and the result is shown in Table S2. The top 30 items in GO enrichment are shown in Figure 2, and their detailed information is shown in Table S3. The key items involved in UFAs metabolism included 3-hydroxyacyl-CoA-dehydrogenase activity (GO: 0003857), unsaturated fatty acid biosynthetic process (GO: 0006636), very-long-chain fatty acid biosynthetic process (GO: 0042761), long-chain fatty acyl-CoA biosynthetic process (GO: 0035338), fatty acid biosynthetic process (GO: 0006633), lipid droplet (GO: 0005811), sphingolipid biosynthetic process (GO: 0030148), protein secretion (GO: 0009306), fatty acid β-oxidation (GO: 0006635), protein binding, bridging (GO: 0030674), protein localization to plasma membrane (GO: 0072659) and calcium ion binding (GO: 0005509). The above items can be mainly classified into fatty acids synthesis, fatty acids transport and signal transduction.

### 3.4. Correlation of UFA Content with Crucial Protein Abundance 

**Figure 3:**
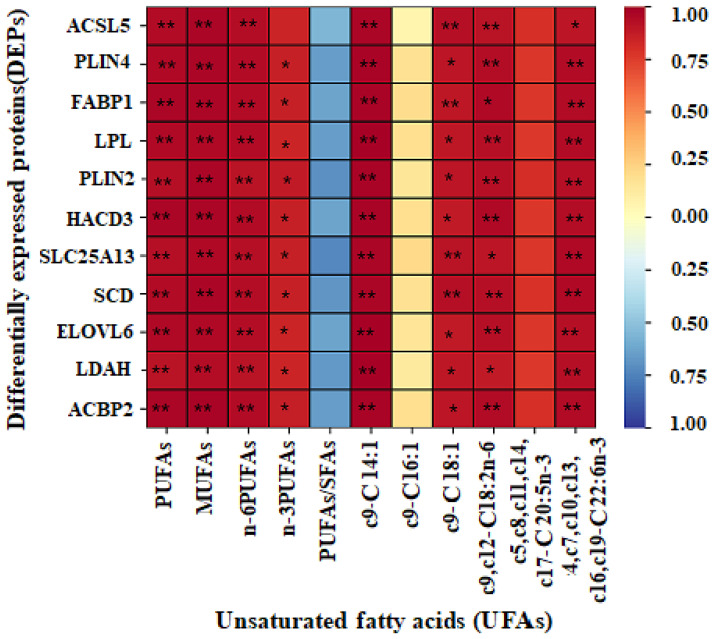
The Pearson correlation of UFAs absolute content, PUFAs/SFAs with the abundance of important proteins in yak subcutaneous fat. Color represents the Pearson’s correlation coefficient. When the color changes from blue to red, the value is −1 to 1. One asterisk showed *p* < 0.05, and two asterisks showed *p* < 0.01. ACSL5: long-chain-fatty acid-CoA ligase 5, PLIN4: perilipin-4, FABP1: fatty acid-binding protein, LPL: lipoprotein lipase, PLIN2: perilipin-2, HACD3: very-long-chain (3R)-3-hydroxyacyl-CoA dehydratase 3, SLC25A13: calcium-binding mitochondrial carrier protein aralar2, SCD: acyl-CoA desaturase, ELOVL6: elongation of very-long-chain fatty acids protein 6, LDAH: lipid droplet-associated hydrolase and ACBP2: acyl-CoA-binding protein.

The result of the Pearson correlation between UFAs absolute content and crucial proteins abundance is shown in Figure 3. The absolute content of c9-C14:1, c9-C18:1, c9, c12-C18:2, c4, c7, c10, c13, c16, c19-C22:6, PUFAs, MUFAs, n-6PUFAs in yak subcutaneous fat was in positive correlation with the abundance of long-chain fatty-acid-CoA ligase 5 isoform X1 (ACSL5), fatty acid-binding protein (FABP1), lipoprotein lipase isoform X1 (LPL), very-long-chain(3R)-3-hydroxyacyl-CoA dehydratase 3 (HACD3), calcium-binding mitochondrial carrier protein aralar2 (SLC25A13), acyl-CoA desaturase (SCD), elongation of very-long-chain fatty acids protein 6 (ELOVL6), lipid droplet-associated hydrolase (LDAH) and acyl-CoA-binding protein isoform X2 (ACBP2) (*p* < 0.05), respectively.

### 3.5. Quantitative Results of DEPs by LC-PRM-MS

**Figure 4:**
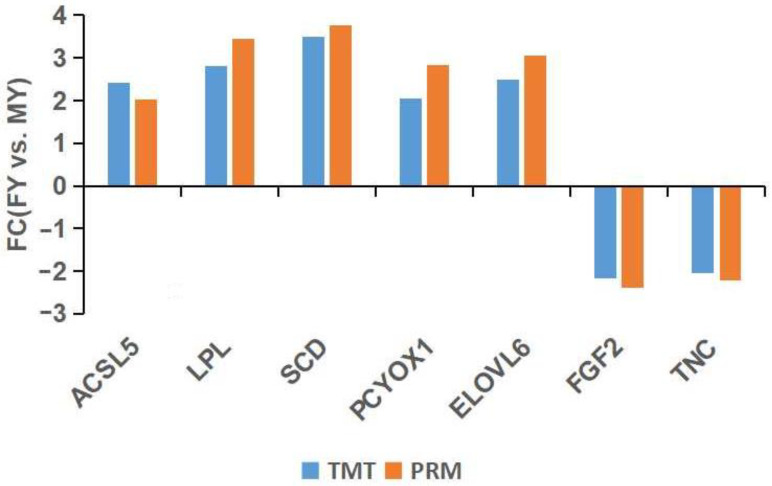
Validation and comparison of fold changes (FCs) in seven DEPs between parallel reaction monitoring (PRM) and the tandem mass tag (TMT) method.

To validate the reproducibility of the DEPs from TMT proteome analysis, ACSL5, LPL, SCD, PCYOX and ELOVL6, fibroblast growth factor 2 (FGF2) and tenascin (TNC) were quantified by LC-PRM-MS analysis. The FCs of the abundance of the above proteins in subcutaneous fat (FYs vs. MYs) by TMT proteome and LC-PRM-MS analysis were compared (Figure 4). LC-PRM-MS analysis showed the abundances of ACSL5, LPL, SCD, PCYOX1 and ELOVL6 in the FYs group were higher, whereas the abundances of FGF2 and TNC in the FYs group were lower. All seven DEPs possessed similar expression patterns in comparison to the TMT data, indicating the reliability of TMT data. The above results indicated the TMT result was accurate and reliable, and can provide a reliable basis for further bioinformatics analysis.

## 4. Discussion

The relative content of MUFAs, UFAs and PUFAs in MY subcutaneous fat was higher than their values in FY subcutaneous fat. The intramuscular fat of bulls had a high relative content of total PUFAs and n-6 PUFAs, too [21]. Therefore, the effect of sex to the relative content of PUFAs in yak and cattle has similar features. Existing research has shown a high value of SFAs/UFAs in animal-origin food can lead to vascular and coronary diseases [22]. Therefore, the consumer needs to increase their PUFAs intake and decrease their SFAs intake, where the recommended value of PUFAs/SFAs is above 0.4 [23]. In this study, the result of fatty acids showed the relative content of MUFAs and PUFAs in subcutaneous fat of MYs were higher, and the value of PUFAs/SFAs was 0.41, so the MY subcutaneous fat was more healthy for the consumer than FY subcutaneous fat. On the other hand, the absolute content of UFAs, MUFAs, PUFAs, n-3 PUFAs and n-6 PUFAs in FY subcutaneous fat was all higher than the values in MY subcutaneous fat. On this basis, a study was conducted to compare FY subcutaneous fat with higher UFAs content (MUFAs and PUFAs) against MY subcutaneous fat with lower UFA content, and then discuss the DEPs associated with UFA content in yak subcutaneous fat. 

The fatty acids in yak adipose tissue come principally from transport in blood and synthesis in adipose cells. LPL is responsible for the hydrolysis of triglycerides in chylomicrons and low-density lipoproteins, and thereby plays an important role in fat clearance from the bloodstream and fat storage, and is considered as the key factor in determining fat content [24]. The Pearson correlation showed the LPL abundance was strongly correlated with MUFA (c9-C14:1, c9-C18:1 and total MUFAs) and PUFA (c9, c12-C18:2, c4, c7, c10, c13, c16, c19-C22:6 and total PUFAs) content in yak subcutaneous fat. High LPL abundance means that more UFAs (MUFAs and PUFAs) were transported into yak adipocytes. On the other hand, fatty acids are elongated and desaturated by enzymes in the endoplasmic reticulum (ER) membrane. A variety of MUFAs can be formed from oleate by elongation and desaturation reactions. The absolute content of total MUFAs, c9-C14:1 and c9-C18:1 in FY subcutaneous fat was higher than those values in MY subcutaneous. In mammals, ELOVL6 catalyzes fatty acids elongation [25], and the high abundance of ELOVL6 increases MUFA content in goat mammary epithelial cells [26] and c5, c8, c11, and c14-C20:4n6 content in bovine adipocytes [27]. SCD (∆9 desaturase) is essential for MUFAs (c9-C18:1, c9-C16:1 and so on) biosynthesis [28]. SCD is a major factor in fatty acid desaturation in growing bovines [29], and is positively correlated with total MUFA content in beef and the c9-C18:1 and total UFA content in milk lipid [30]. In this study, ELOVL6 and SCD abundance were in strong positive correlation with MUFA (c9-C14:1, c9-C18:1 and total MUFAs) content in yak subcutaneous fat, so the above two proteins can be seen to play a key role in the MUFA synthesis in yak subcutaneous fat. 

PUFAs can not be directly biosynthesized in livestock, and must be derived from precursors PUFAs [31]. Current research indicates that n-6 PUFAs, such as arachidonic acid in mammals, are synthesized by linoleic acid, while n-3 PUFAs, such as c4, c7, c10, c13, c16, c19-C22:6 and c5, c8, c11, c14, and c17-C20:5 in mammals, are synthesized by c9, c12, and c15-C18:3 [32]. In this study, c9, c12-C18:2, c5, c8, c11, c14, c17-C20:5n, c4, c7, c10, c13, c16, c19-C22:6, PUFA and n-6 PUFA content in FY subcutaneous fat was higher. The enzymes involved in n-3 UFAs interconversion are analogous to the n-6 UFAs pathway for C18:2 transferring to C20:4, and mainly include ACBPs and HACDs. Long-chain fatty acids (LCFAs) can be further converted to very-long-chain fatty acids (VLCFAs) by endoplasmic reticulum (ER) membrane-bound enzymes cycling through a similar four-step process, and the HACDs are responsible for the dehydration [33]. A high abundance of HACD3 promotes PUFAs elongation [34]. ACBP is a small primarily cytosolic protein that binds acyl-CoA esters with high specificity, and modulates the donation of acyl-CoA esters for β-oxidation and complex PUFAs synthesis [35]. The studies of ACBP on fatty acid transport focus on ACBP3 and ACBP4 at present [36], and the function of ACBP2 has not been reported. In this study, the abundance of HACD3 and ACBP2 in yak subcutaneous fat was in positive correlation with c9, c12-C18:2, c4, c7, c10, c13, c16, c19-C22:6, PUFA, n-6 PUFA and n-3 PUFA content, so the PUFAs synthesis in yak subcutaneous fat is promoted by the high abundance of HACD3 and ACBP2. 

UFAs transfer into triglyceride by esterification. ACSLs are key enzymes regulating the import/export system. Different ACSL members have similar functional protein structural units and coenzyme A binding sites [37]. ACSL1 is widely studied and is involved in fatty acids oxidation, fatty acids transport and triglyceride synthesis [38]. It has been reported that ACSL1 promotes fat synthesis in bovine adipocytes [39], but ACSL5 function on bovine fat is still unclear at present. FABP has been assumed to be adipose-tissue specific and used as an adipocyte marker, and promotes fat content in cattle by acting as intracellular transport of hydrophobic intermediates and fat metabolites through the membranes [40]. The high abundance of FABP1 significantly increases fatty acid uptake in the hepatocyte [41]. The abundance of ACSL5 and FABP1 were in positive correlation with c9-C14:1, c9-C18:1, c9, c12-C18:2, c4, c7, c10, c13, c16, c19-C22:6, total PUFA, total MUFA and total n-6PUFA content in yak subcutaneous fat, and the high abundance of ACSL5 and FABP1 indicates that more triglyceride deriving from UFAs (PUFAs and MUFAs) is synthesized in yak adipocyte.

**Figure 5:**
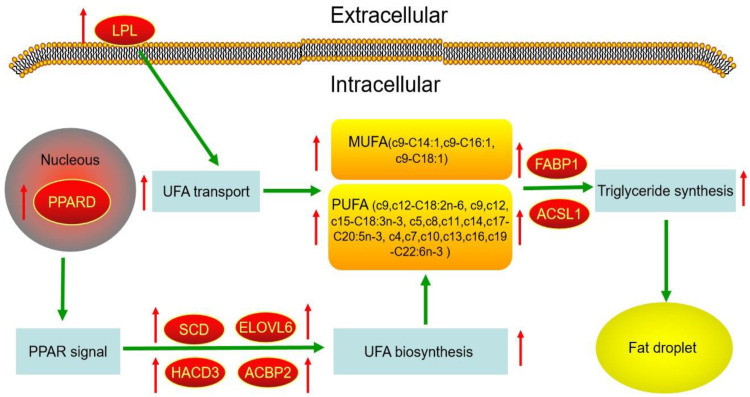
The potential regulatory network of UFAs metabolism according to the DEPs. Green arrows indicate the possible regulatory relationships. Box represents the metabolic process and UFAs. Red oval represents the crucial protein in UFAs metabolism. Red arrow represents the upregulated expression, and green arrow represents relevance or consequence.

Peroxisome proliferator-activated receptor (PPAR) is a master regulator of adipogenesis [42], and its activation produces the upregulation of genes implicated in fatty acids transport and binding. At present, three major isoforms of PPAR (PPARA, PPARD and PPARG) have been identified [43]. PPARG plays a vital role in the abundance of many proteins related to fat homeostasis [44], and regulates fatty acid synthesis and transport in cattle [45], but there is no report on the PPARD regulating fatty acids metabolism in cattle. In this study, the abundance of PPARG in FY and MY subcutaneous fat kept the same level, but the abundance of PPARD was upregulated (FC = 1.89, *p* < 0.05) in FY subcutaneous fat with high UFA, MUFA and PUFA content. PPARD and PPARG possess a similar function in the regulation of fat deposition in mammals, so it was inferred that the regulation of UFA and PUFA content in yak subcutaneous fat was achieved by the transcription factor PPARD and downstream proteins SCD, ELOVL6, LPL, FABP1, HACD3 and ACBP2 (Figure 5). The UFAs and PUFAs transport and synthesis in yak subcutaneous fat can be increased by the high abundance of the above proteins. Moreover, these downstream proteins in the PPAR signal are the potential biomarkers for high UFA and PUFA content in yak subcutaneous fat. In the future, the following method may be tried during the development of new yak breeds with high UFA and PUFA content: a small quantity of subcutaneous fat samples in calves is collected by biopsy sampling, and the abundance of above biomarker proteins is detected by Western blotting (WB). The individuals with high protein abundances will be chosen and carried out the next generation breeding. Compared with the early lactation, there was an increase in the concentrations of PUFA in milk from the late lactation, and this regulation was realized by PPARG, FABP3, FABP4 and so on in the mammary tissue of cows [46]. The UFA content in the muscle of cattle is closely connected with ACSL1 [47] and PPAR [4]. Therefore, the regulation of UFA content in yak possesses some unique properties, and is different from cattle and cow in some ways. 

## 5. Conclusions

In conclusion, this study gives a better understanding of proteome differences in subcutaneous fat of yaks of different sex. UFA, PUFA, MUFA, n-3PUFA and n-6PUFA content in FY subcutaneous fat is higher than those values in MY subcutaneous fat, whereas the MY subcutaneous fat is more healthy for the consumer. The regulation of UFA and PUFA content in the subcutaneous fat of yaks of different sex is initiated by the transcription factor PPARD. The increase in c9, c12-C18:2n-6, c9, c12, c15-C18:3n-3, c5, c8, c11, c14, c17-C20:5n-3, c4, c7, c10, c13, c16, c19-C22:6n-3, and total PUFA content in yak subcutaneous fat is realized by the high abundance of LPL, FABP1, HACD3, ACSL5 and ACBP2 in PPAR signaling. The bioinformatics analysis further shows that the above five DEPs play a crucial role in UFA transport and synthesis, and can be recognized as potential biomarkers for UFAs, especially PUFA content in yak subcutaneous fat. The above results provide a theoretical basis for further studies on the regulation of UFA and PUFA content in yak subcutaneous fat.

## Figures and Tables

**Table 1:** The common nutrition and main fatty acid content of grass (dry matter).

Nutrition Component	Content (Mean ± SD, g/100 g)
Ash	7.66 ± 0.06
Crude fat	2.63 ± 0.20
Crude protein	11.93 ± 0.40
Neutral detergent fiber	76.14 ± 0.79
Acid detergent fiber	10.09 ± 0.62
Calcium	5.22 ± 0.32
Phosphorus	0.07 ± 0.002
C16:0	0.30 ± 0.05
C18:0	0.09 ± 0.02
c9-C18:1	0.07 ± 0.01
c9, c12-C18:2n6	0.29 ± 0.05
c9, c12, c15-C18:3n3	0.68 ± 0.11

c = cis. SD: standard deviation.

**Table 2:** The absolute and relative content of fatty acids in the subcutaneous fat of female yaks (FYs, *n* = 6) and male yaks (MYs, *n* = 6).

Variable	Absolute Content (Mean ± SD, g/100 g Subcutaneous Fat)		Relative Content (Mean ± SD, g/100 g of Total Fatty Acids)	
	FYs	MYs	FYs	MYs
C4:0	0.17 ± 0.05	0.18 ± 0.08	0.24 ± 0.06	0.31 ± 0.14
C6:0	0.03 ± 0.01	0.03 ± 0.01	0.04 ± 0.02	0.05 ± 0.02
C8:0	0.004 ± 0.001	0.003 ± 0.001	0.005 ± 0.001	0.006 ± 0.001
C10:0	0.02 ± 0.01	0.02 ± 0.01	0.03 ± 0.008	0.03 ± 0.010
C11:0	0.003 ± 0.001	0.003 ± 0.001	0.004 ± 0.002	0.004 ± 0.002
C12:0	0.02 ± 0.003	0.02 ± 0.002	0.02 ± 0.003	0.03 ± 0.004
C13:0	0.10 ± 0.03	0.07 ± 0.05	0.14 ± 0.04	0.12 ± 0.08
C14:0	0.35 ± 0.06	0.32 ± 0.06	0.50 ± 0.08	0.54 ± 0.10
C14:0iso	0.05 ± 0.02	0.05 ± 0.02	0.07 ± 0.02	0.08 ± 0.03
c9-C14:1	0.12 ± 0.01	0.02 ± 0.01 **	0.17 ± 0.02	0.04 ± 0.01 **
C15:0	0.20 ± 0.03	0.19 ± 0.03	0.28 ± 0.03	0.31 ± 0.04
C15:0iso	0.17 ± 0.05	0.18 ± 0.04	0.24 ± 0.07	0.30 ± 0.07
C15:0ai	0.07 ± 0.01	0.09 ± 0.01 *	0.10 ± 0.02	0.14 ± 0.03 *
c10-C15:1	0.07 ± 0.01	0.07 ± 0.01	0.10 ± 0.01	0.12 ± 0.03
C16:0	11.08 ± 1.44	8.46 ± 0.99 **	15.83 ± 1.72	14.10 ± 1.61
C16:0iso	0.15 ± 0.02	0.18 ± 0.01 *	0.21 ± 0.04	0.30 ± 0.03 **
c9-C16:1	0.93 ± 0.05	0.82 ± 0.10 *	1.33 ± 0.07	1.36 ± 0.14
C17:0	0.30 ± 0.08	0.37 ± 0.12	0.43 ± 0.10	0.61 ± 0.16 *
C17:0iso	0.16 ± 0.03	0.18 ± 0.03	0.23 ± 0.04	0.30 ± 0.04 *
C17:0ai	0.25 ± 0.03	0.28 ± 0.06	0.36 ± 0.05	0.47 ± 0.11 *
c10-C17:1	0.12 ± 0.03	0.13 ± 0.08	0.17 ± 0.04	0.22 ± 0.12
C18:0	21.93 ± 0.99	17.92 ± 0.98 **	31.41 ± 0.75	29.86 ± 0.67 **
C18:0iso	0.13 ± 0.03	0.14 ± 0.02	0.18 ± 0.04	0.23 ± 0.04 *
c9-C18:1	13.43 ± 0.32	12.30 ± 0.32 **	19.22 ± 0.20	20.51 ± 0.62 **
t11-C18:1	0.42 ± 0.08	0.45 ± 0.07	0.59 ± 0.11	0.75 ± 0.13 *
c9, c12-C18:2n-6	9.22 ± 0.28	8.07 ± 0.37 **	13.20 ± 0.33	13.44 ± 0.40
c9, t11-C18:2	0.23 ± 0.09	0.21 ± 0.04	0.33 ± 0.13	0.34 ± 0.07
t11, c15-C18:2	0.70 ± 0.23	0.70 ± 0.18	1.00 ± 0.31	1.17 ± 0.33
c6, c9, c12-C18:3n-6	0.14 ± 0.07	0.14 ± 0.07	0.20 ± 0.06	0.23 ± 0.06
c9, c12, c15-C18:3n-3	0.54 ± 0.04	0.43 ± 0.04 **	0.77 ± 0.05	0.72 ± 0.07
c9, t11, c15-C18:3	0.05 ± 0.01	0.05 ± 0.01	0.08 ± 0.01	0.08 ± 0.01
C20:0	0.05 ± 0.01	0.04 ± 0.02	0.07 ± 0.01	0.07 ± 0.03
c11-C20:1	0.28 ± 0.07	0.22 ± 0.07	0.41 ± 0.11	0.37 ± 0.11
c11, c14-C20:2	0.06 ± 0.01	0.06 ± 0.02	0.09 ± 0.01	0.10 ± 0.03
C21:0	0.41 ± 0.12	0.35 ± 0.08	0.59 ± 0.18	0.58 ± 0.14
c5, c8, c11, c14-C20:4n-6	2.94 ± 0.33	2.62 ± 0.34	4.22 ± 0.53	4.37 ± 0.50
c11, c14, c17-C20:3n-3	0.002 ± 0.001	0.003 ± 0.002	0.003 ± 0.001	0.005 ± 0.002 *
c5, c8, c11, c14, c17-C20:5n-3	0.63 ± 0.04	0.55 ± 0.05 *	0.89 ± 0.04	0.92 ± 0.08
C22:0	0.10 ± 0.05	0.08 ± 0.05	0.14 ± 0.06	0.14 ± 0.08
C23:0	0.11 ± 0.03	0.11 ± 0.04	0.16 ± 0.04	0.18 ± 0.06
C24:0	2.62 ± 0.52	2.41 ± 0.20	3.75 ± 0.79	4.01 ± 0.36
c4, c7, c10, c13, c16, c19-C22:6n-3	0.021 ± 0.002	0.016 ± 0.002 **	0.031 ± 0.002	0.027 ± 0.003 *
c11-C24:1	1.51 ± 0.26	1.45 ± 0.38	2.17 ± 0.38	2.42 ± 0.64
SFAs	38.44 ± 1.26	31.67 ± 0.92 **	55.04 ± 0.77	52.79 ± 0.54 **
UFAs	31.40 ± 0.49	28.32 ± 0.56 **	44.96 ± 0.70	47.21 ± 0.50 **
MUFAs	16.87 ± 0.24	15.47 ± 0.19 **	24.15 ± 0.26	25.81 ± 0.52 **
PUFAs	14.53 ± 0.34	12.84 ± 0.52 **	20.81 ± 0.33	21.41 ± 0.31 *
n-3PUFAs	1.19 ± 0.08	1.00 ± 0.07 **	1.70 ± 0.09	1.08 ± 0.13
n-6PUFAs	12.30 ± 0.27	10.83 ± 0.64 **	17.61 ± 0.62	18.04 ± 0.70
BCFAs	0.97 ± 0.09	1.09 ± 0.11	1.39 ± 0.14	1.82 ± 0.20 **
BHIs	1.40 ± 0.30	1.40 ± 0.28	2.00 ± 0.40	2.34 ± 0.51
n-6/n-3PUFAs	10.38 ± 0.81	10.83 ± 1.10	10.38 ± 0.81	10.83 ± 1.10
PUFAs/SFAs	0.38 ± 0.01	0.41 ± 0.01 **	0.38 ± 0.01	0.41 ± 0.01 **

c = cis; t = trans. * Significantly different from female yaks (FYs) (*p* < 0.05). ** Significantly different from FYs (*p* < 0.01). SFAs: saturated fatty acids, MUFAs: monounsaturated fatty acids, PUFAs: polyunsaturated fatty acids, BCFAs: branched-chain fatty acids, BHIs: biohydrogenation intermediates.

**Table 3:** Crucial differentially expressed proteins (DEPs) involved in unsaturated fatty acid (UFAs) metabolism in yak subcutaneous fat.

Accession	Description	*p*	FC
XP_014333755.1	Perilipin-4 (PLIN4)	1.27 × 10^−8^	2.10
XP_005910864.1	Acyl-CoA-binding protein isoform X2 (ACBP2)	1.27 × 10^−8^	2.10
XP_005896308.1	Hydroxyacyl-coenzyme A dehydrogenase (HADH)	1.32 × 10^−6^	2.24
XP_005906639.1	Prostaglandin reductase 1 (PTGR1)	1.12 × 10^−6^	2.10
XP_005900453.1	Acetyl-CoA acetyltransferase (ACAT2)	3.19 × 10^−6^	2.19
XP_005896285.1	Elongation of very-long-chain fatty acids protein 6 (ELOVL6)	2.90 × 10^−6^	2.48
XP_005888723.1	Long-chain-fatty-acid-CoA ligase 5 isoform X1(ACSL5)	3.44 × 10^−4^	2.19
XP_005900434.1	Fatty acid-binding protein (FABP1)	8.07 × 10^−6^	2.25
XP_005895719.2	Lipid droplet-associated hydrolase (LDAH)	7.78 × 10^−5^	2.68
XP_014335786.1	Lipoprotein lipase isoform X1 (LPL)	2.74 × 10^−7^	2.81
XP_005895544.1	Serum amyloid A protein-like (SAA1)	5.51 × 10^−10^	4.11
XP_014339229.1	Nucleoside diphosphate kinase (NME4)	5.39 × 10^−6^	2.14
XP_005902329.1	Perilipin-2 isoform X1 (PLIN2)	2.35	2.02
XP_005899116.1	Polyprenol reductase (SRD5A3)	4.07 × 10^−3^	2.22
XP_005900036.1	Very-long-chain (3R)-3-hydroxyacyl-CoA dehydratase 3 (HACD3)	9.56 × 10^−6^	2.09
XP_005887189.1	Calcium-binding mitochondrial carrier protein Aralar2 (SLC25A13)	8.17 × 10^−5^	2.18
XP_005892117.1	Acyl-CoA desaturase (SCD)	3.85 × 10^−6^	3.50
XP_005911054.1	Peroxisome proliferator-activated receptor delta isoform X1	7.69 × 10^−6^	1.89

FC: fold change.

## Data Availability

The mass spectrometry proteomics data have been deposited to the ProteomeXchange Consortium (http://proteomecentral.proteomexchange.org) via the iProX partner repository [48] with the dataset identifier PXD030642 (accessed on 18 October 2021).

## Supplementary Materials

The following supporting information can be downloaded at: https://www.mdpi.com/article/10.3390/genes13050790/s1. Table S1: Information of all differentially expressed protein (DEPs) in female (FYs) and male yaks (MYs) subcutaneous fat. Table S2: Information of all terms in GO enrichment for DEPs in FYs and MYs subcutaneous fat. Table S3: Information of top 30 items in GO enrichment for DEPs in FYs and MYs subcutaneous fat. Figure S1: Chromatogram of fatty acids in subcutaneous fat of FYs. Figure S2: Chromatogram of fatty acids in subcutaneous fat of MYs.
