# Supplementary figures and images for: Explaining Unsaturated Fatty Acids (UFAs), Especially Polyunsaturated Fatty Acid (PUFA) Content in Subcutaneous Fat of Yaks of Different Sex by Differential Proteome Analysis
Source: Genes (Basel). 2022 Apr 28;13(5):790. doi: 10.3390/genes13050790 (PMC9140874; doi:10.3390/genes13050790)

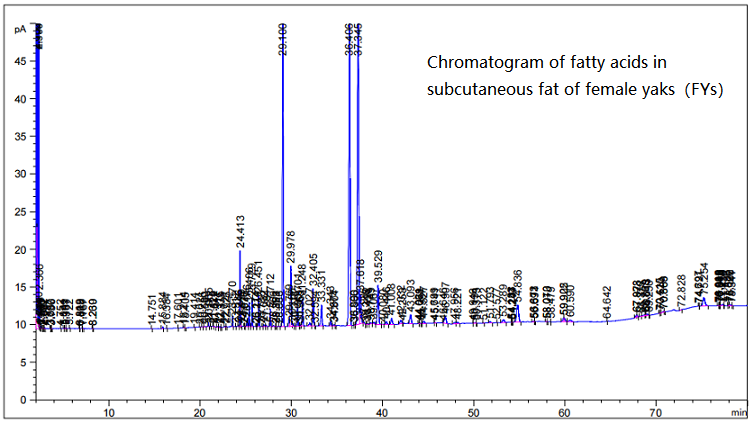

Supplement: Supplementary file 1 [file genes-13-00790-s001.zip › supplementary files/Figure S1.png]

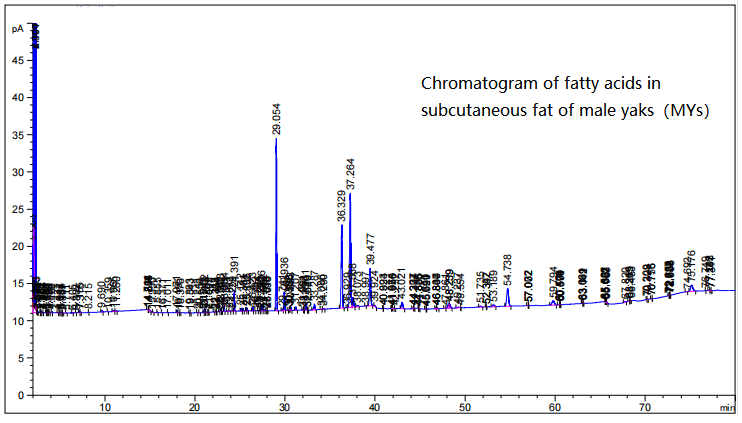

Supplement: Supplementary file 1 [file genes-13-00790-s001.zip › supplementary files/Figure S2.png]
